# Severe COVID-19 in the intensive care unit: a case series 

**DOI:** 10.1186/s13256-021-02799-1

**Published:** 2021-05-03

**Authors:** Hori Hariyanto, Corry Quando Yahya, Ronald Christian Agustinus Aritonang

**Affiliations:** 1 Faculty of Medicine, Department of Anesthesiology and Intensive Care, Universitas Pelita Harapan, Jl. M. H. Thamrin Boulevard 1100, Lippo Village Tangerang, Tangerang, Banten 15811 Indonesia; 2 Faculty of Medicine, Universitas Indonesia, Jalan Diponegoro No 77, Jakarta Pusat, 10430 Indonesia; 3 Siloam Hospitals Kelapa Dua, Jl. Kelapa Dua Raya No.1001, Kelapa Dua, Tangerang, Banten 15810 Indonesia

**Keywords:** Septic shock, COVID-19, ICU, Mechanical ventilation, Severe infection, Respiratory failure, Case report

## Abstract

**Background:**

Coronavirus disease 2019 (COVID-19) was first identified in Indonesia in March 2020, and the number of infections has grown exponentially. The situation is at its worst, overwhelming intensive care unit (ICU) resources and capacity.

**Case presentation:**

This is a single-center observational case study of 21 confirmed COVID-19 patients admitted to the ICU from March 20, 2020, to April 31, 2020. Demographics, baseline comorbidities, clinical symptoms, laboratory tests, electrocardiogram (ECG) and chest imaging were obtained consecutively during patient care. We identified 21 patients with confirmed COVID-19 severe infection in our ICU. The mean (± standard deviation) age of the patients was 54 ± 10 years; 95% were men, with shortness of breath (90.6%) the most common symptom. Hypertension was identified as a comorbidity in 28.6% of patients. The most common reason for admission to the ICU was hypoxemic respiratory failure, with 80% (17 patients) requiring mechanical ventilation. Half of the patients (10) died between day 1 and day 18, with septic shock as the primary cause of death. Of the 11 surviving patients, five were discharged home, while six were discharged from the ICU but remained in the hospital ward. Even then, the median length of ICU stay amongst survivors was 18 days.

**Conclusions:**

To date, there are no known effective antiviral agents or specific therapy to treat COVID-19. As severe systemic inflammatory response and multiple organ failure seems to be the primary cause of death, supportive care in maintaining oxygenation and hemodynamic stability remain the mainstay goals in treating critically ill COVID-19 patients.

## Background

Coronavirus disease 2019 (COVID-19) has spread from a single city to the entire globe with alarming speed. Arising from China, this virus has expanded rapidly to all parts of the world, knowing no geopolitical boundaries in infecting the human population. The first case of COVID-19 in Indonesia was identified in March 2020. Since then, the number of cases in Indonesia has grown exponentially; as of October 7, 2020, there had been 315,714 confirmed COVID-19 cases and 11,472 deaths [1]. While most patients with COVID-19 are asymptomatic or experience only mild symptoms, some individuals develop acute respiratory distress syndrome (ARDS) requiring mechanical ventilation, while some succumb to septic shock. Reports describing patients admitted to the intensive care unit (ICU) in Indonesia are sparse; therefore, it is our aim to share our early experience of COVID-19 pandemic care amongst ICU patients.

## Case presentation

### Study design and participants

This is a single-center observational case series study. All patients completed an informed consent form that was approved by the Ethical Committee at Siloam Hospital Kelapa Dua (Study protocol: 19-03-0317). Data were collected consecutively during admission. Enrollment included all patients admitted to the ICU starting with the first patient in March 20, 2020 up to April 31, 2020. All 21 cases enrolled in this study were confirmed COVID-19 from double-gene polymerase chain reaction (PCR) detection of the severe acute respiratory syndrome coronavirus 2 (SARS-CoV-2) using a nasopharyngeal swab in line with the diagnostic criteria guideline established by the Indonesian Ministry of Health.

### Data collection

Demographics, baseline comorbidities, clinical symptoms, laboratory tests, chest imaging and electrocardiogram (ECG) changes were obtained consecutively during patient visits to the ICU. Diagnoses during the hospital course, inpatient medications, treatments including invasive mechanical ventilation and kidney replacement therapy, and outcomes including length of stay, discharge and mortality were also recorded. To quantify the extent of infection, a severity score was calculated using the CURB-65 [confusion, urea, respiratory rate, blood pressure, and 65 years of age or older] pneumonia risk score and Acute Physiology Assessment and Chronic Health Evaluation II (APACHE II) score.

### Statistical analysis

Variables are reported as frequency, percentage (%), mean (SD) if they were normally distributed, and median with range (min–max) for non-normal distribution. Laboratory results are presented as actual data, and all data analysis was carried out using STATA version 12 software (StataCorp LLC, College Station, TX, USA).

## Results

### Patient characteristics

During the period from March 20, 2020, through April 31, 2020, we identified 21 critically ill patients with confirmed COVID-19 infection admitted to the ICU. The demographic and clinical characteristics of the patients are shown in Table 1. The mean (± SD) age of the patients was 54 ± 10 years (range 31–79); 20 (95%) were male and one (4.8%) was female. The mean duration of symptoms before hospital admission was 8 ± 3 days. All patients were Indonesian citizens of Malay ethnicity, and none had recently traveled to a country with known transmission such as China, South Korea, Iran or Italy. However, the majority of patients confirmed recent contact exposure from various cluster sites including family and religious gatherings. Comorbidities of patients in this critically ill population included diabetes 1 (4.8%), hypertension 6 (28.6%) and cerebrovascular disease 1 (4.8%). One (4.8%) patient was documented to be a former smoker, and another patient (4.8%) had chronic obstructive pulmonary disease.

**Table 1:** Demographics and baseline characteristics of patients with severe COVID-19

Variables	All patients (*N* = 21)
Age (years)	
Mean ± SD	54 ± 10
Range	31–79
Gender	
Male	20 (95.2%)
Female	1 (4.8%)
Duration of symptoms (days)	
Mean ± SD	8 ± 3
Travel history	
Yes	0 (0%)
No	21(100%)
Comorbidities	
Diabetes	1 (4.8%)
Hypertension	6 (28.6%)
Cerebrovascular disease	1 (4.8%)
Smoking	1 (4.8%)
Chronic obstructive pulmonary disease	1 (4.8%)
Presenting symptoms	
Fever	18 (85.7%)
Cough	18 (85.7%)
Shortness of breath	19 (90.4%)
Fatigue	3 (14.2%)
Sore throat	2 (9.5%)
Myalgia	2 (9.5%)
APACHE II score	
10–14	7 (33.3%)
15–19	10 (47.6%)
20–24	2 (9.5%)
> 25	2 (9.5%)
CURB-65 pneumonia risk score	
0	9 (42.9%)
1	9 (42.9%)
2	3 (14.3%)

Data are median (IQR), *n* (%) or *n*/*N* (%), where *N* is the total number of patients with available data

*SD* standard deviation, *IQR* interquartile range, *ICU* intensive care unit, *APACHE II* Acute Physiology and Chronic Health Evaluation II, *CURB-65* confusion, urea, respiratory rate, blood pressure, and 65 years of age or older

Symptoms presented upon admission included fever [18 (85.7%) of 21 patients], cough [18 (85.7%)] and shortness of breath [19 (90.4%)]. Other symptoms reported were fatigue [3 (14.2%)], sore throat [2 (9.5%)] and myalgia [2 (9.4%)]. Upon admission, the mean APACHE score was 10–14 in seven patients (33.3%), 15–19 in 10 (47.6%), 20–24 in two (9.5%) and greater than 25 in two (9.5%). The mean CURB-65 score was 0 was nine patients (42.9%); 1 in nine patients (42.9%) and 2 in three patients (14.3%).

In this study, all patients received hydroxychloroquine, azithromycin, meropenem and antifungal prophylaxis; eight patients (38%) received compassionate-use tocilizumab, and no patients received systemic steroids. Thromboprophylaxis was given with heparin 250 U/hour, intravenously.

### Laboratory findings

Table 2 shows the laboratory and radiologic findings of patients upon admission to the ICU. On admission, lymphocytopenia was common (in 86% of the patients), with a mean leukocyte count of 11.056 ± 6.604 × 10^3^/μL and low median lymphocyte count of 13.5% (interquartile range 1–19%). Inflammation markers including erythrocyte sedimentation rate (ESR), C-reactive protein (CRP) and lactate dehydrogenase were also measured, and all values were dramatically elevated. Mean lactate dehydrogenase was uniformly elevated at 951 ± 140, along with mean CRP level of 217 ± 122. Hepatic alanine aspartate enzyme was 40 U/L or higher in all patients.

**Table 2:** Laboratory data at admission to the intensive care unit and imaging findings

Variables	All patients (*n* = 21)
Leukocyte count (10^3^/µL)	
Mean ± SD	11.056 ± 6.604
Lymphocytes (%)	
Median (IQR)	13.5 (1–19)
Lactate dehydrogenase (U/L)	
Mean ± SD	951 ± 140
C-reactive protein (mg/L)	
Mean ± SD	217 ± 122
Alanine aspartate (U/L)	40
Chest radiograph	
Bilateral pulmonary opacities	21 (100%)
Pleura effusion	12 (57.1%)
Computed tomography (CT) scan: bilateral ground glass opacities and consolidation	6 (29%)

Data are median (IQR), *n* (%), or *n*/*N* (%), where *N* is the total number of patients with available data

*SD* standard deviation, *IQR* interquartile range

**Fig. 1:**
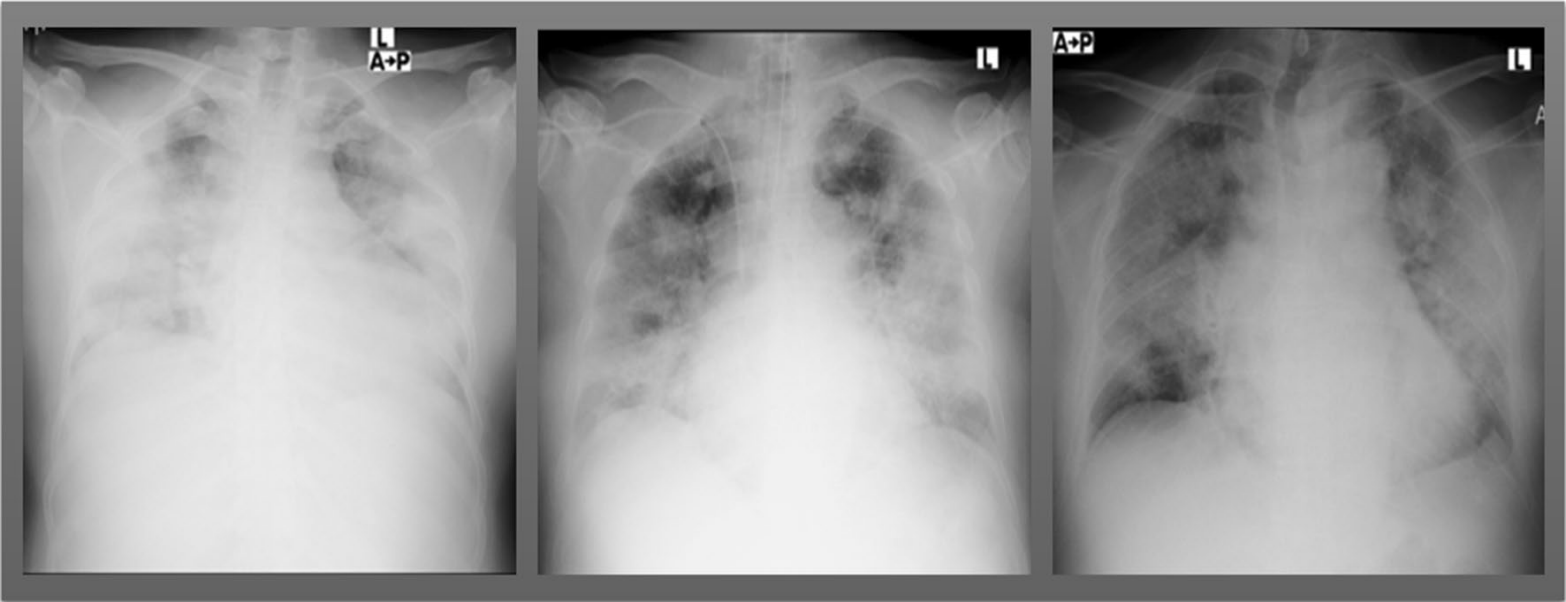
Chest films of severe COVID-19 patients upon admission to the intensive care unit

Chest radiographs were obtained in all 21 patients, all of which showed bilateral pulmonary opacities, while pleural effusion was seen in 12 (57.1%) of the patients (Fig. 1). A computed tomography (CT) scan of the chest was obtained in six patients (29%); five of which showed bilateral ground glass opacities and one consolidation. Overall, 17 patients progressed to respiratory distress and required mechanical ventilation, while the other four were discharged to the ward after a mean of 13 days in the ICU.

### Respiratory failure

Seventeen patients (80.9%) received invasive mechanical ventilation, as their ratios of arterial oxygen partial pressure to fraction of inspired oxygen [PaO_2_:FiO_2_ (p/f ratio)] were consistent with severe acute respiratory distress syndrome (ARDS): mean p/f ratio 100 ± 36. The time to initiation of mechanical ventilation was 4 ± 3 days, and all patients were placed in the prone position starting day 2 of mechanical ventilation.

The median FiO_2_ on day 1 of mechanical ventilation was 0.9 (interquartile range 0.7–1.0); on day 3, median FiO_2_ was 0.6 (interquartile range 0.5–0.7), and on day 5 median FiO_2_ was 0.4 (interquartile range 0.35–0.55). The median driving pressure [the difference between plateau pressure and positive end-expiratory pressure (PEEP)] on day 1 of mechanical ventilation was 23 ± 5 cmH_2_O, with median pulmonary compliance of 20 mL/cmH_2_O (interquartile range, 13–27). Initial PEEP was set at 11 ± 2 cmH_2_O. Throughout 5 days of mechanical ventilation, the median driving pressure was gradually lowered to 15 ± 3 cmH_2_O, pulmonary compliance improved to 42 mL/cmH_2_O (interquartile range, 28–52), and PEEP was maintained at 9 ± 1 cmH_2_O. The mean p/f ratio was 150 ± 62 on day 1, 193 ± 112 on day 3, and 235 ± 109 on day 5. Out of 17 patients, two (13%) developed progressive ARDS and died. Seven (41%) patients survived, with a mean duration of mechanical ventilation of 10 ± 4.8 days. Amongst these, one underwent bronchoscopy due to atelectasis; three encountered pneumothorax, and two underwent tracheostomy due to difficulty in weaning and prolonged mechanical ventilation support (greater than 20 days of mechanical ventilation).

### Shock

Twelve patients (75%) presented with concurrent hypotension requiring vasopressors without clear evidence of secondary infection. Of these patients, three (18%) had transient hypotension after intubation; nine (56%) had hypotension that was unrelated to intubation or that persisted for more than 12 hours after intubation. Six patients (38%) developed septic shock and died; one (6%) experienced cardiac arrest upon prone positioning, and another patient (6%) experienced cardiac arrest due to intractable hyperkalemia and persistent acidosis, despite undergoing hemodialysis.

### Outcomes

**Fig. 2:**
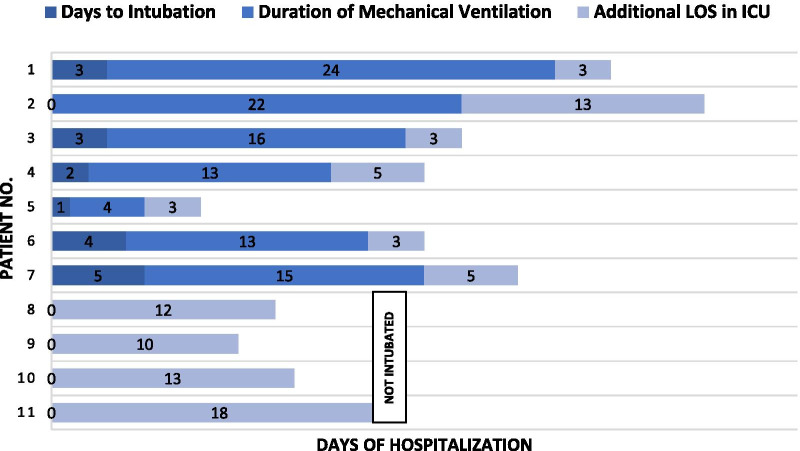
Duration of therapy amongst 11 intensive care unit survivors of severe COVID-19. *LOS* length of stay

As of May 31, out of the 21 patients cared for in the ICU, 10 (47%) had died and 11 survived, with six (23%) patients who had been discharged from the ICU but remained in the hospital and five (23%) who had been discharged from the hospital (Fig. 2). The median length of ICU stay among survivors was 18 days (interquartile range, 7–36), while the median length of ward stay after ICU discharge was 11 days (interquartile range, 7–25). Fitness for discharge was based on the absence of fever for at least 7 days, improvement in chest radiograph and negative nasopharyngeal PCR test.

## Discussion and conclusion

The majority of patients admitted to our ICU were men, with a mean age of 54 ± 10 years, and had hypertension as a comorbidity. Clinical manifestations were fever, cough and shortness of breath. No gastrointestinal, renal or cerebrovascular manifestations were documented in our study. All 21 patients had abnormal blood test results with elevated CRP and liver enzymes, decreased lymphocytes, increased D-dimer and coagulation abnormalities, all of which were similar to reports from China [2, 3]. Six chest CT scans were performed showing ground-glass opacities and/or consolidation similar to other reports [4].

Recent studies have highlighted two phenotypes in COVID-19 pneumonia. The L-type lung is characterized by normal compliance, low ventilation-to-perfusion ratio and low lung weight. Over time, the lungs may either improve or evolve into an H-type pneumonia characterized by low compliance, high right-to-left shunt and increasing pulmonary edema, which contribute to the deadly cycle of hypoxemia and strain on body organs [5]. In this report, the majority [18 (85.8%)] of the 21 patients had an admission CURB score of 0–1. Nevertheless, more than half progressed to severe ARDS and respiratory failure as evidenced by hypoxemia, progressive bilateral infiltrates and decreased respiratory system compliance (H-type COVID-19 pneumonia). Out of 17 patients receiving mechanical ventilation, two rapidly progressed to severe ARDS and died.

High-flow nasal cannula was initially used to improve oxygenation, but promptly escalated to mechanical ventilation once increased work of breathing was observed. Notably, high oxygen requirements and poor lung compliance were observed soon after initiation of mechanical ventilation. In severe ARDS, damage to type II alveolar cells not only renders surfactant inactive, but these edematous alveoli also compress alveoli in dependent regions, thereby contributing to alveolar collapse [6]. Prone positioning has the benefit of reopening collapsed alveoli, as the heart rests on the sternum and exerts less pressure on the pleura and lung [7]. This together with the lung recruitment maneuver opens the dorsal parts of the lung and allows more homogeneous ventilation and perfusion [8]. Therefore, a high initial PEEP (10-12 cmH_2_O) was given and patients were placed in the prone position for 6 hours per day. Prone positioning started on day 2 of mechanical ventilation, and an increased p/f ratio was observed from day 3 onwards.

Early in the clinical course, sputum production was minimal and sterile. As mechanical ventilation continued, coexisting lower respiratory bacterial infections were identified, further complicating the course of disease and resulting in longer ICU stays. Seven patients survived, with two encountering pneumothorax and placed on tracheostomy due to prolonged ventilator support, while the other five were successfully liberated from mechanical ventilation without any long-term sequelae. Even then, the median ICU stay among the survivors was a lengthy 18 days (interquartile range, 7–36 days).

In this study, all 21 patients received hydroxychloroquine, azithromycin, meropenem and antifungal prophylaxis, with eight patients (38%) receiving compassionate-use tocilizumab. Unfortunately, one of our patients experienced Torsades de pointes and died. Such fatal arrhythmia may have been caused by the direct effect of hydroxychloroquine and azithromycin on ventricular repolarization, thus prolonging the QT interval [9]. Hence, hydroxychloroquine and azithromycin use was terminated halfway through the course of ICU care. None of our patients received steroids, as studies during that time were inconclusive for the use of systemic glucocorticoids.

Upon admission, the majority of our patients had an APACHE score of 10–19 (mortality score of 12–22%); nevertheless, six (35%) of 17 patients who received mechanical ventilation died due to septic shock. Symptoms were similar to septic shock caused by bacterial infections, but one distinctive difference was the deterioration that occurred within a very short time (< 24 hours). This might be attributable to the massive explosive release of viral antigens, thus creating a violent inflammatory response and sudden hemodynamic collapse, as others have speculated [10, 11]. Taken together, this suggests that no severity scores seem to aid in predicting the future course and prognosis of COVID-19 infection.

To date, there are still no solid markers for predicting disease progression, and various treatments with immunomodulators, antivirals and interleukin inhibitors are given with hopes of halting the progression of the disease, but no consensus guidelines have yet been developed. To make matters worse, this virus possesses remarkable mimicry capability, as it displays atypical presentation ranging from gastrointestinal symptoms, neurologic complications, antiphospholipid syndrome and acute myocardial injury to fatal ventricular arrhythmia [12–15], all of which may lead to a false diagnosis, delay treatment and postpone isolation measures within a community.

COVID-19 has emerged as a complex disease that appears to have many “faces.” Despite evidence of extensive damage both in radiologic and laboratory findings, the clinical presentation does not always seem to conform. In the midst of this pandemic, we would like to share our experience of caring for those with the greatest severity of illness: the ICU population. We understand the limitations of our study relating to its small sample size and limited laboratory investigations. However, our experience in caring for these patients has reminded us that supportive therapy remains the hallmark in fighting this self-limiting disease. Until new evidence becomes available, physicians can expect mechanical ventilation to be a lengthy journey, with bacterial co-infections, sepsis and pneumothorax encountered along the course of ICU stay.

## Data Availability

Please contact the author for data requests.

## Abbreviations

COVID-19: Coronavirus disease 2019
ICU: Intensive care unit
SD: Standard deviation
WHO: World Health Organization
ARDS: Acute respiratory distress syndrome
PCR: Polymerase chain reaction
CURB-65: Confusion, urea, respiratory rate, blood pressure, and 65 years of age or older pneumonia risk score
APACHE II: Acute Physiology and Chronic Health Evaluation II
ESR: Erythrocyte sedimentation rate
CRP: C-Reactive protein
CT: Computed tomography
p/f: PaO_2_:FiO_2_
FiO_2_: Fraction of inspired oxygen
PEEP: Positive end-expiratory pressure
